# Inertia and Decision Making

**DOI:** 10.3389/fpsyg.2016.00169

**Published:** 2016-02-16

**Authors:** Carlos Alós-Ferrer, Sabine Hügelschäfer, Jiahui Li

**Affiliations:** Department of Economics, University of CologneCologne, Germany

**Keywords:** inertia, decision making, Bayesian updating, multiple processes, perseveration, preference for consistency

## Abstract

Decision inertia is the tendency to repeat previous choices independently of the outcome, which can give rise to perseveration in suboptimal choices. We investigate this tendency in probability-updating tasks. Study 1 shows that, whenever decision inertia conflicts with normatively optimal behavior (Bayesian updating), error rates are larger and decisions are slower. This is consistent with a dual-process view of decision inertia as an automatic process conflicting with a more rational, controlled one. We find evidence of decision inertia in both required and autonomous decisions, but the effect of inertia is more clear in the latter. Study 2 considers more complex decision situations where further conflict arises due to reinforcement processes. We find the same effects of decision inertia when reinforcement is aligned with Bayesian updating, but if the two latter processes conflict, the effects are limited to autonomous choices. Additionally, both studies show that the tendency to rely on decision inertia is positively associated with preference for consistency.

## 1. Introduction

As described in Newtonian physics, the term “inertia” refers to the fact that, in the absence of external resistance, a moving object will keep moving in the same direction. This word has also been used across multiple fields as a metaphor to describe related characteristics of human behavior. For example, in management and organization science, the expression “cognitive inertia” describes the phenomenon that managers might fail to reevaluate a situation even in the face of change (Huff et al., 1992; Reger and Palmer, 1996; Hodgkinson, 1997; Tripsas and Gavetti, 2000). In medical studies, “therapeutic inertia” or “clinical inertia” describe the failure of health care providers to intensify therapy when treatment goals are unattained (Phillips et al., 2001; Okonofua et al., 2006). In sociology, “social inertia” depicts the resistance to change or the (excess) stability of relationships in societies or social groups (Bourdieu, 1985). In psychology, the “inertia effect” describes individuals' reluctance to reduce their confidence in a decision following disconfirming information (Pitz and Reinhold, 1968). The concept of “psychological inertia” has been proposed to describe the tendency to maintain the status-quo (Gal, 2006). Suri et al. (2013) speak of “patient inertia” to describe the phenomenon that many patients stick to inferior options or fail to initiate treatment even after the diagnosis of a medical problem.

Summing up, the concept of inertia has been used to describe many different phenomena related to a resistance to change. The existence of these phenomena has been linked to status-quo bias (Samuelson and Zeckhauser, 1988; Ritov and Baron, 1992), described as the tendency to maintain the defaults either by repeating a decision or avoiding action. So far, however, our understanding of the processes underlying inertia in decision making is rather limited. In the present study, we aim to contribute to this understanding by focusing on a particular facet of inertia, which we term “decision inertia:” the tendency to repeat a previous choice, regardless of its outcome, in a subsequent decision. We investigate whether this tendency significantly influences active decision making and explore the psychological processes behind it using a belief-updating task.

The phenomenon we explore here is consistent with previous evidence from the decision-making literature. For instance, Pitz and Geller (1970) observed a tendency to repeat previous decisions even following disconfirming information. In a study on reinforcement in belief-updating tasks, which was not focused on inertia, Charness and Levin (2005) nevertheless observed a “taste for consistency,” corresponding to the phenomenon that people were prone to repeat their choices, no matter whether these choices led to success or failure. In a study on perceptual decision making, Akaishi et al. (2014) showed that choices tend to be repeated on subsequent trials, even on the basis of little sensory evidence. Erev and Haruvy (in press) review studies on decision making from experience where, for instance, participants repeatedly choose between a risky prospect and a safe option, and receive immediate feedback (e.g., Nevo and Erev, 2012). Erev and Haruvy (in press) conclude that there exists a strong tendency to simply repeat the most recent decision, which is even stronger than the tendency to react optimally to the most recent outcome. Furthermore, Zhang et al. (2014) showed that the tendency to repeat previous decisions exists even for unethical behavior. There might also be a relation to the extensive literature on choice-induced preference change, which shows that earlier decisions alter preferences, and hence might result in repeated choices (see also Ariely and Norton, 2008; Sharot et al., 2010; Alós-Ferrer et al., 2012).

The influence of previous decisions on subsequent choices has also been investigated in reinforcement learning research. For instance, Lau and Glimcher (2005) studied trial-by-trial behavior of monkeys in a matching task in which the reward structure favored alternating between two choice options. They observed that choosing a particular alternative decreased the probability of choosing that alternative again on the next trial, but increased the likelihood of choosing it again some time in the future, regardless of reward history. Studies in which participants worked on probabilistic “bandit tasks” that favored sticking with successful options showed that participants were prone to repeat their choices, independently of any effects due to previous rewards (e.g., Schönberg et al., 2007, Supplemental Results). Accordingly, reinforcement learning models now account for the influence of past choices on subsequent ones by including a model parameter of “perseveration,” capturing the tendency to repeat or avoid recently chosen actions (e.g., Schönberg et al., 2007; Gershman et al., 2009; Wimmer et al., 2012; for an introduction to model-based reinforcement learning, see Daw, 2012). The inclusion of such a parameter leads to more accurate predictions in contrast to models that merely incorporate the effect of past reinforcers (see Lau and Glimcher, 2005).

To understand decision inertia, we consider a multiple-process framework (Evans, 2008; Sanfey and Chang, 2008; Weber and Johnson, 2009; Alós-Ferrer and Strack, 2014), that is, we consider individual decisions as the result of the interaction of multiple decision processes. Specifically, we follow the assumptions of parallel-competitive structured process theories, which propose that multiple processes affect behavior simultaneously, resulting in conflict or alignment among these processes (e.g., Epstein, 1994; Sloman, 1996; Strack and Deutsch, 2004). Whenever several decision processes are in conflict (i.e., deliver different responses), cognitive resources should be taxed, resulting in longer response times and higher error rates. These predictions were confirmed in a response-times study by Achtziger and Alós-Ferrer (2014), which showed that more errors arise and responses are slower when Bayesian updating (i.e., normatively optimal behavior) is opposed to reinforcement learning of the form “win-stay, lose-shift.” We relied on a variant of the experimental paradigms employed in Achtziger and Alós-Ferrer (2014), Achtziger et al. (2015), and Charness and Levin (2005) but focused on the conflict with decision inertia, viewed as a further decision process. We measured error rates and response times to investigate the role of decision inertia in a belief-updating task. Specifically, we hypothesized that decision inertia is a further process potentially conflicting with optimal behavior and affecting decision outcomes and decision times. Accordingly, our main hypotheses were that more errors and slower choices would be made in cases of conflict between decision inertia and Bayesian updating.

To further explore decision inertia, we considered possible individual correlates of this decision process. We hypothesized that decision inertia would be associated with preference for consistency (PFC), which is a desire to be and look consistent within words, beliefs, attitudes, and deeds, as measured by the scale with the same name (Cialdini et al., 1995). Cialdini (2008) argues that because of the tendency to be consistent, individuals fall into the habit of being automatically consistent with previous decisions. Once decision makers make up their minds about a given issue, consistency allows them to not think through that issue again, but leads them to fail to update their beliefs in the face of new information when confronting new but similar decision situations. Furthermore, Pitz (1969) observed that inertia in the revision of opinions is the result of a psychological commitment to initial judgments. Thus, we hypothesized that preference for consistency might be one of the possible mechanisms driving decision inertia, in which case an individual's behavioral tendency to rely on decision inertia should be positively associated with their preference for consistency (PFC).

Our last hypothesis concerns the kind of decisions leading to decision inertia. If this phenomenon arises from a tendency to be consistent with previous decisions, and hence economize decision costs, the effect should be stronger following autonomous decisions (free choices) than required ones (forced choices). The same prediction also arises from a different perspective. In general, human decision makers prefer choice options that they freely chose over options with equal value that they did not choose, as exemplified by the literature on choice-induced preference change (e.g., Lieberman et al., 2001; Sharot et al., 2009, 2010; Alós-Ferrer et al., 2012). Relying on behavioral and genotype data, Cockburn et al. (2014) recently investigated the underlying mechanism of this preference. In a probabilistic learning task, their participants demonstrated a bias to repeat freely chosen decisions, which was limited to rewarded vs. non-rewarded decisions. Interindividual differences in the magnitude of this choice bias were predicted by differences in a gene that has been linked to reward learning and striatal plasticity. Cockburn et al. (2014) interpret these findings as evidence that free choices selectively amplify dopaminergic reinforcement learning signals, based on the workings of a feedback loop between the basal ganglia and the midbrain dopamine system. Given such an amplification of the value of freely chosen options, it again follows that decision inertia should be more pronounced after autonomous decisions compared to forced ones in our study. We make use of the fact that the standard implementation of the paradigms we rely on includes both forced and free choices to test this hypothesis.

## 2. Study 1

### 2.1. Methods

#### 2.1.1. Experimental design

Decision making under uncertainty or risk requires integrating different pieces of information on the possible outcomes in order to form and update probability judgments (beliefs). From a normative point of view, the correct combination of previous (prior) beliefs on the probability of an uncertain event and additional information is described by Bayes' rule (Bayes and Price, 1763). The present study used a two-draw decision paradigm (Charness and Levin, 2005; Achtziger and Alós-Ferrer, 2014; Achtziger et al., 2015), where of course Bayesian updating is the rational strategy to derive optimal decisions. There are two urns, the Left Urn and the Right Urn, each containing 6 balls, which can be black or white. The urns are presented on the computer screen, with masked colors for the balls. Participants are asked to choose which urn a ball should be extracted from (with replacement) by pressing one of two keys on the keyboard, and are paid for drawing balls of a predefined color, say black (the winning ball color is counterbalanced across participants). After observing the result of the first draw, participants are asked to choose an urn a second time, a ball is again randomly extracted, and they are paid again if the newly extracted ball is of the appropriate color. The payment per winning ball in our implementation was 18 Euro cents.

The urn composition (i.e., number of black and white balls) in Study 1 is given in Table 1 (left-hand side). The essence of the design is that the composition varied according to a “state of the world,” Up or Down, which was not revealed to participants. That is, participants knew the urn compositions in each state of the world and the fact that those were held constant for the whole experiment. They were also informed that the prior probability of each state was 1/2. Further, they knew that the state of the world was constant within the two-draw decision, but was randomized according to the prior for each new round. This means that the first draw is uninformed, but by observing the first ball's color the decision maker can draw conclusions about the most likely state of the world. Thus, for a second draw, an optimizer should choose the urn with the highest expected payoff, given the posterior probability of the state of the world updated through Bayes' rule. Given the urn compositions in Study 1, straightforward computations show that an optimizer should stay with the same urn as in the first draw after a win and switch after a loss. For example, if a black ball is extracted from the Left Urn, the updated probability of being in the state “Up” is (1/2)(2/6)/((1/2)(2/6) + (1/2)(4/6)) = 1/3, hence choosing the Left Urn again delivers an expected payoff of (1/3)(2/6) + (2/3)(4/6) = 5/9, while switching to the Right Urn delivers a smaller expected payoff of (1/3)(4/6) + (2/3)(2/6) = 4/9. Note that optimizing behavior given this particular urn composition is fully aligned with that prescribed by an intuitive reinforcement rule (win-stay, lose-shift); we will return to this point in Study 2. Decision inertia, on the other hand, prescribes to always stay with the same urn as in the first draw, independently of whether that decision resulted in a win or a loss. Hence, Bayesian updating conflicts with decision inertia after drawing a losing ball in the first draw.

**Table 1:** **Urn composition in Studies 1 and 2**.

	**Study 1**			**Study 2**	
State (Prob)	Left Urn	Right Urn	State (Prob)	Left Urn	Right Urn
Up (1/2)	● ● ○ ○ ○ ○	● ● ● ● ○ ○	Up (1/2)	● ● ● ● ○ ○	● ● ● ● ● ●
Down (1/2)	● ● ● ● ○ ○	● ● ○ ○ ○ ○	Down (1/2)	● ● ○ ○ ○ ○	○ ○ ○ ○ ○ ○

*An unknown state of the world (Up, Down) with a known prior 1/2 determines the composition of two urns (Left, Right) containing six balls each (black or white). All winning balls paid the same independently of which urn they came from*.

Participants repeated the two-draw decision 60 times. Following Charness and Levin (2005) and Achtziger and Alós-Ferrer (2014), we included both forced first draws (where the choice is dictated to the participant) and free first draws. This also allows us to explore the effect of decision inertia arising from previous autonomous choices as opposed to required choices. To avoid confounding forced choices and learning effects, participants made forced draws and free draws alternately.

#### 2.1.2. Participants

Participants were recruited using ORSEE (Greiner, 2004), a standard online recruitment system for economic experiments which allows for random recruitment from a predefined subject pool. Participants were students from the University of Cologne. 45 participants (29 female; age range 18–32, mean 23.51) participated in exchange for performance-based payment plus a show-up fee of 2.50 Euros. Three further participants had to be excluded from data analysis due to technical problems (missing data).

#### 2.1.3. Procedure

The experiment was conducted at the Cologne Laboratory for Economic Research (CLER) using z-Tree (Fischbacher, 2007). Experimental procedures were in accordance with the ethical standards laid down in the 1964 Declaration of Helsinki and its later amendments, and also standard practices in experimental economics (e.g., no-deception rule). In agreement with the ethics and safety guidelines at the CLER, participants were all pre-registered in the laboratory through ORSEE and had given written informed consent regarding the laboratory's guidelines (no further informed consent is necessary for particular experiments). Potential participants were informed of their right to abstain from participation in the study or to withdraw consent to participate at any time without reprisal. Participants were randomly assigned to the two counterbalance conditions (winning ball color). Before the start of the experiment, participants read instructions and answered control questions to ensure they understood the experiment properly. Then the experimental task started, which lasted around 10 min. After the task, participants filled in questionnaires including the Preference for Consistency scale (brief 9-item version, continuously ranging from 0 to 10; Cialdini et al., 1995) and demographic questions. A session lasted about 1 h and average earnings were 13.85 Euros (*SD* = 1.02).

### 2.2. Results

#### 2.2.1. Error rates

**Figure 1:**
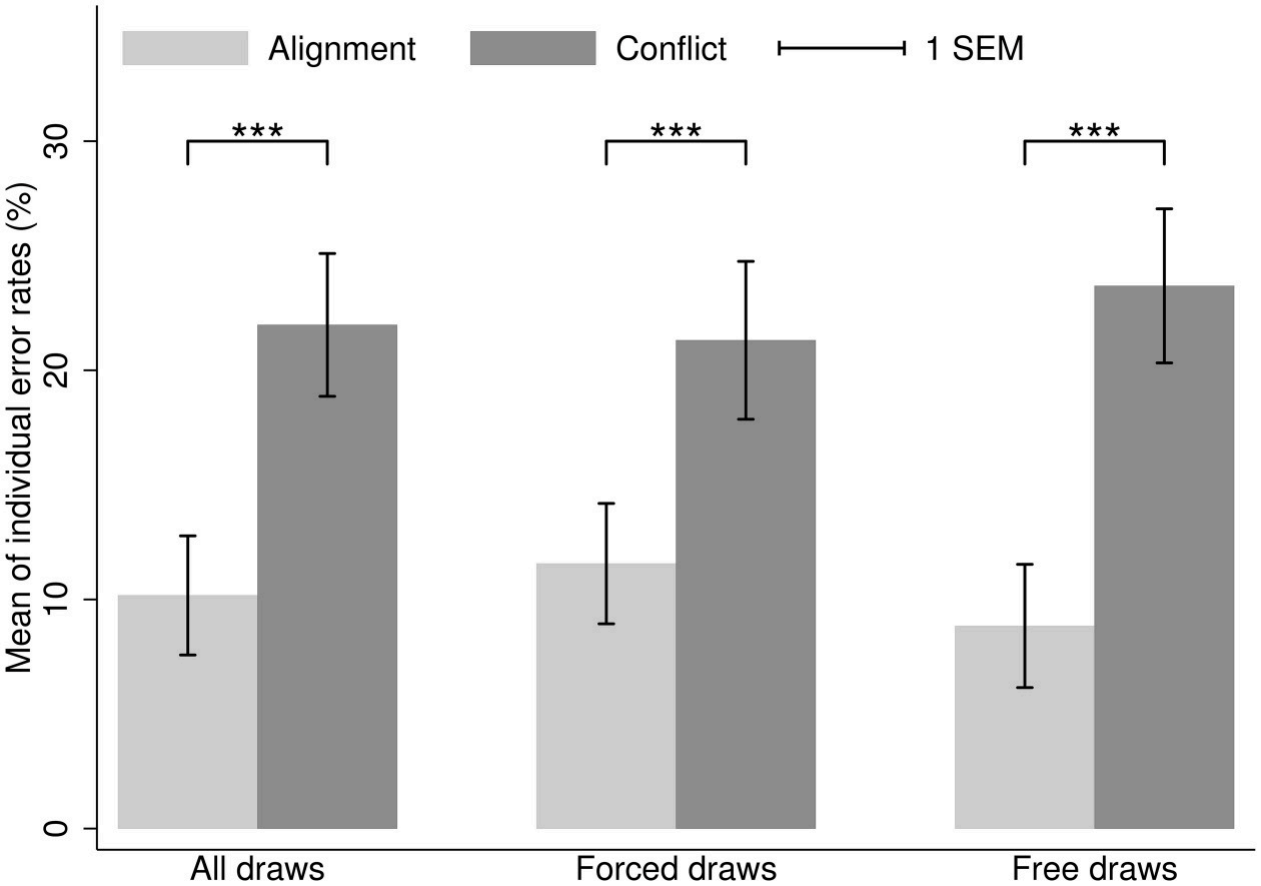
**Study 1**. Mean of individual error rates in case of alignment (light gray) and conflict (dark gray) between Bayesian updating and inertia. Error bars represent standard errors. ^***^p < 0.01.

Mean error rates are depicted in Figure 1. The mean error rate in case of conflict between inertia and Bayesian updating was 21.98% (*SD* = 20.91%), vs. just 10.18% (*SD* = 17.43%) in case of alignment. To test for differences in the distribution of individual-level error rates, here and elsewhere in the paper we rely on non-parametric, two-tailed Wilcoxon Signed-Rank tests (WSR). The difference is highly significant (median error rate 15.63% in case of conflict, 5.26% in case of alignment; *N* = 45, *z* = 3.79, *p* < 0.001). When we split the tests conditional on forced draws and free draws, the result holds both for forced draws (median error rate 14.29% in case of conflict (mean 21.31%, *SD* = 23.12%), 6.67% in case of alignment (mean 11.56%, *SD* = 17.62%); WSR test, *z* = 2.89, *p* = 0.004) and free draws (median error rate 17.65% in case of conflict (mean 23.68%, *SD* = 22.56%), 0% in case of alignment (mean 8.84%, *SD* = 18.03%); WSR test, *z* = 3.94, *p* < 0.001). Paired *t*-tests provide similar results, but since error rates are not normally distributed we favor WSR tests, which are ordinal in nature. We rely on standard WSR tests that adjust for zero differences, but results are highly similar when using WSR tests that ignore zero differences. Furthermore, to test the robustness of the WSR results, we additionally ran a two-way ANOVA (Factor 1: conflict with inertia vs. alignment with inertia; Factor 2: forced draw vs. free draw) on log-transformed error rates. Since several participants had error rates of 0% (which is commonly observed in the present paradigm), we used the log(*x* + 1) transformation following Bartlett (1947) to be able to deal with zero values. The ANOVA results were consistent with the results based on the WSR test, showing a significant main effect of conflict with vs. alignment with inertia, but no main effect of forced vs. free draw and no interaction.

#### 2.2.2. Response times

Second-draw responses were significantly longer in case of conflict with inertia (median 973 ms, mean 1119 ms, *SD* = 447 ms) than in case of alignment (median 903 ms, mean 1001 ms, *SD* = 319 ms). We tested the difference in distributions with a WSR test on individual average response times (*N* = 45, *z* = 2.13, *p* = 0.033). However, the result only holds for free draws (conflict: median 933 ms, mean 1048 ms, *SD* = 478 ms; alignment: median 787 ms, mean 840 ms, *SD* = 289 ms; *z* = 3.54, *p* < 0.001), but not for forced draws (conflict: median 1038 ms, mean 1191 ms, *SD* = 478 ms; alignment: median 1069 ms, mean 1179 ms, *SD* = 440 ms; *z* = −0.06, *p* = 0.951). We further ran a two-way ANOVA (Factor 1: conflict with inertia vs. alignment with inertia; Factor 2: forced draw vs. free draw) on log-transformed response times (since the distribution of response times was skewed). Results were consistent with the WSR test, showing significant main effects of both factors and a significant interaction effect.

### 2.3. Discussion

The results of Study 1 support the idea that decision inertia corresponds to an automatic process conflicting with Bayesian updating, thereby affecting decision performance and decision times. In particular, more errors were made when Bayesian updating and decision inertia delivered different responses. Additionally, after free draws decisions were significantly slower when Bayesian updating and decision inertia were opposed compared to when they were aligned. However, this decision-times evidence of a decision conflict was not observed after forced draws, suggesting that the effect of decision inertia might be stronger following voluntary choices.

## 3. Study 2

Study 2 investigated decision inertia in a more complex setting, where more than two decision processes are in conflict. Previous studies (Charness and Levin, 2005; Achtziger and Alós-Ferrer, 2014; Achtziger et al., 2015) have shown that in probability-updating paradigms, reinforcement processes (cued by winning or losing in the first draw) play a relevant role. Reinforcement roughly corresponds to the psychological Law of Effect: the propensity to adopt an action increases when it leads to a success and decreases when it leads to a failure (Thorndike, 1911; Sutton and Barto, 1998). Charness and Levin (2005) introduced the “reinforcement heuristic” as a decision rule, defined as a simple “win-stay, lose-shift” behavioral principle which might give different prescriptions than Bayesian updating and thereby produce errors. In fact, Charness and Levin (2005) observed error rates above 50% when the heuristic conflicted with Bayes' rule, which demonstrates that reinforcement has a significant impact on individuals' decision making. By analyzing response times, Achtziger and Alós-Ferrer (2014) showed that reinforcement is a rather automatic process conflicting with the more controlled process of Bayesian updating. In Study 1, due to the distribution of balls in the two urns, Bayesian updating and reinforcement were always aligned, and hence our analysis could not be confounded by a conflict with reinforcement. In Study 2 we aimed to test if inertia still plays a role when reinforcement additionally conflicts with Bayesian updating. We used the same experimental paradigm as in Study 1, but with a different urn composition, resulting in two kinds of (endogenous) decision situations. In the first kind, Bayesian updating and reinforcement were aligned, allowing for a conceptual replication of Study 1. In the second kind, there was a conflict between Bayesian updating and reinforcement.

### 3.1. Method

#### 3.1.1. Experimental design

The experimental task differs from Study 1 only (but crucially) in the urn composition, which is shown in Table 1 (right-hand side). Given this composition, choosing the Right Urn in the first draw reveals the state of the world and the decision for the second draw is straightforward, i.e., win-stay, lose-shift. That is, as in Study 1, both Bayesian updating and the reinforcement heuristic give the same prescription, but decision inertia conflicts with Bayesian updating after drawing a losing ball from this urn. Choosing the Left Urn in the first draw leads to a different situation. Given the posterior probability updated through Bayes' rule, Bayesian updating prescribes to stay after a loss and to switch after a win (win-shift, lose-stay), which is opposed to the prescriptions of reinforcement. Further, Bayesian updating conflicts with inertia after drawing a winning ball (but not after drawing a losing ball). Thus, after starting with Left there are situations where reinforcement and decision inertia are aligned and both conflict with Bayesian updating.

#### 3.1.2. Participants and procedure

Forty-four participants (25 female; age range: 19–31, mean 23.80) were recruited using the same enrollment method, experimental procedures, and payment rules as in Study 1. Average earnings were 14.29 Euros (*SD* = 0.78). Four further participants had to be excluded from data analysis due to technical problems.

### 3.2. Results

#### 3.2.1. Error rates

**Figure 2:**
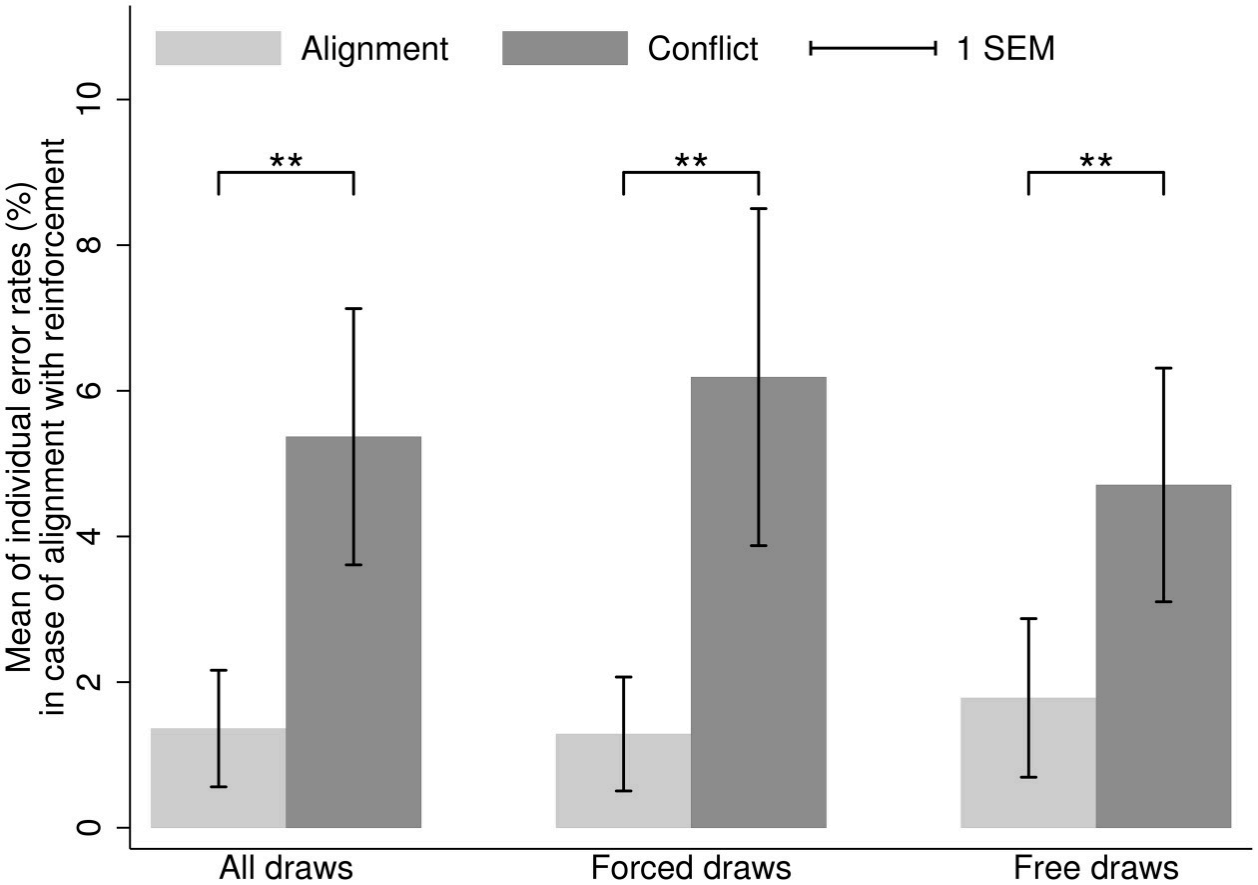
**Study 2**. Mean of individual error rates in case of alignment (light gray) and conflict (dark gray) between Bayesian updating and inertia, for the situations where Bayesian updating is aligned with reinforcement (first draw from the Right Urn). Error bars represent standard errors. ^**^p < 0.05.

**Figure 3:**
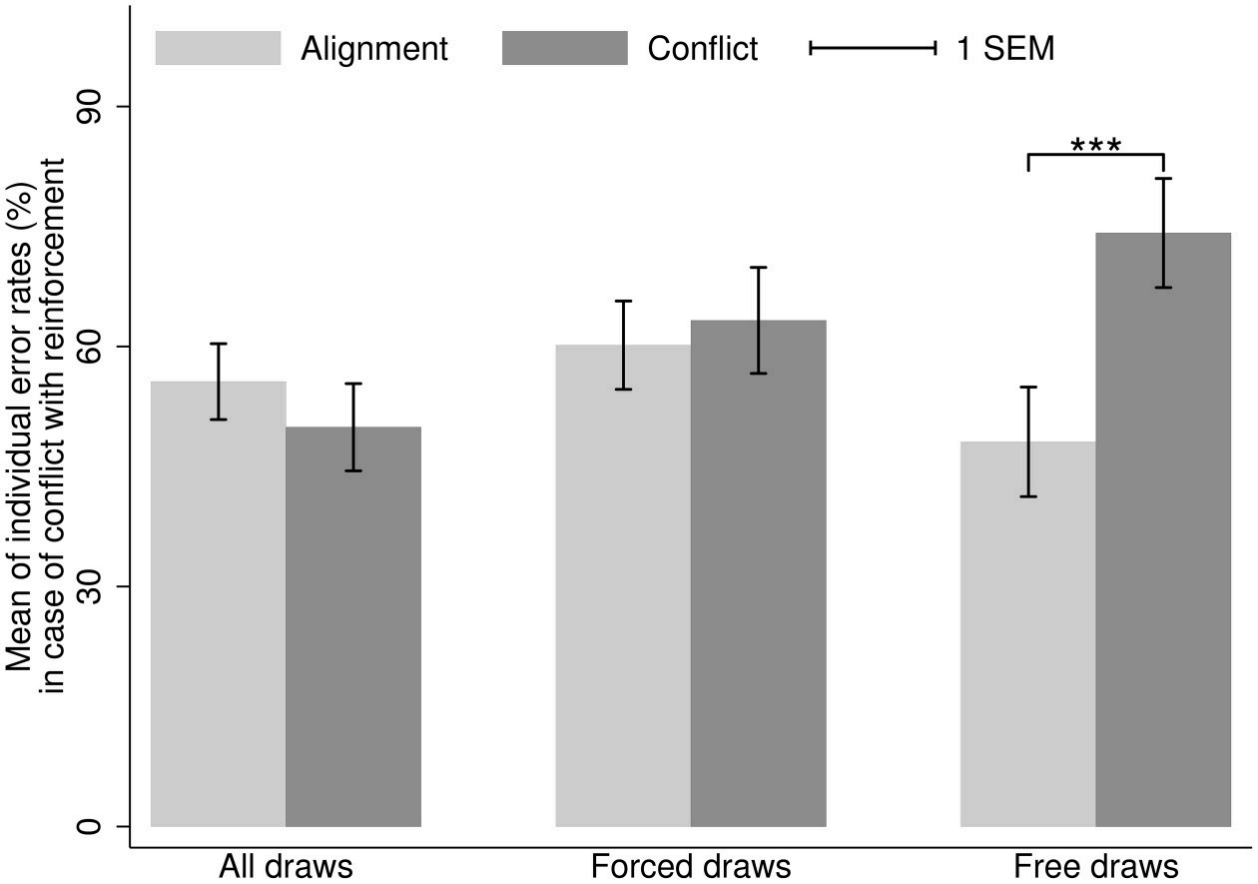
**Study 2**. Mean of individual error rates in case of alignment (light gray) and conflict (dark gray) between Bayesian updating and inertia, for the situations where Bayesian updating conflicts with reinforcement (first draw from the Left Urn). Error bars represent standard errors. ^***^p < 0.01.

Figures 2, 3 depict the means of individual-level error rates depending on the type of draws in Study 2. The results for situations with no conflict between Bayesian updating and reinforcement (the Right Urn situations; Figure 2) were analogous to those of Study 1. In this case, error rates are naturally very low, because reinforcement learning prescribes the correct answer (e.g., Achtziger and Alós-Ferrer, 2014). In situations where Bayesian updating conflicts with reinforcement (the Left Urn situations; Figure 3), error rates were considerably higher than under alignment with reinforcement. The observed error rates are highly similar to those found in previous studies using the same decision task (Charness and Levin, 2005; Achtziger and Alós-Ferrer, 2014; Achtziger et al., 2015). The high error rates can be explained by the automaticity of the reinforcement process, which seems to be highly dominant for some participants. Interestingly, when looking at individual data, one observes only few error rates in the 50% range, but rather one cluster of participants with error rates below 1/3, and another cluster with rates above 2/3. This points to interindividual heterogeneity (see Achtziger et al., 2015), but speaks against the possibility that participants responded randomly.

Turning back to our hypotheses regarding decision inertia, consider first decision situations where Bayesian updating and reinforcement are aligned. In this case, the mean error rate in case of conflict between decision inertia and Bayesian updating (further supported by reinforcement) was 5.37% (*SD* = 11.67%), while in case of alignment between those two processes (hence alignment among all three processes) it was only 1.36% (*SD* = 5.31%). Although all medians were at a 0% error rate, the difference in distributions was significant (WSR test, *N* = 44, *z* = 2.57, *p* = 0.010). This result holds both for forced draws (conflict: median 0%, mean 6.19%, *SD* = 15.17%; alignment: median 0%, mean 1.29%, *SD* = 5.13%; WSR test, *N* = 43, *z* = 2.21, *p* = 0.027) and free draws (conflict: median 0%, mean 4.71%, *SD* = 10.52%; alignment: median 0%, mean 1.78%, *SD* = 7.14%; WSR test, *N* = 43, *z* = 2.31, *p* = 0.021). Note that for free draws *N* is reduced due to a participant who avoided starting with the Right Urn when first draws were free, and hence provided no data for this particular comparison. We also excluded this participant from the corresponding analysis for forced draws to ensure that the subset of participants for the analysis of both draw types was the same. As in Study 1, we additionally ran a two-way ANOVA on the transformed error rates as a robustness check. The pattern of results was consistent with the WSR tests, showing a significant main effect of conflict with vs. alignment with inertia, but no main effect of forced vs. free draw and no interaction.

Consider now the situations where Bayesian updating conflicts with reinforcement (the Left Urn situations; Figure 3). In this case, error rates for the cases of conflict and alignment of Bayesian updating with decision inertia were similar (mean 49.92% (*SD* = 36.15%) and 55.61% (*SD* = 31.44%), respectively). The difference was not significant (median in case of conflict 48.81%, in case of alignment 62.02%; WSR test, *N* = 44, *z* = −0.86, *p* = 0.391). If we consider only free draws, as we expected, there are more errors in case of conflict between Bayesian updating and inertia (mean 74.19%, *SD* = 34.08%) than in case of alignment (mean 48.08%, *SD* = 34.23%). The difference in distributions is significant (median in case of conflict 90%, in case of alignment 50%; WSR test, *N* = 25, *z* = 2.73, *p* = 0.006). The extraordinarily high error rates in case of conflict are revealing but intuitive, for in this case the correct normative response is opposed to positive reinforcement, that is, inertia actually prescribes to merely repeat a successful choice. Note that in this case the test needs to exclude the participants who avoided starting with Left when first draws were free, and hence provided no data for this particular comparison (indeed, this possibility is the original reason for including forced draws in the design). Again, we also excluded these participants from the corresponding analysis for forced draws. If we consider only forced draws, however, the difference of error rates between the case of conflict with inertia and the case of alignment with inertia is not significant (medians 66.67% in case of conflict (mean 63.26%, *SD* = 33.13%), 66.67% in case of alignment (mean 60.16%, *SD* = 27.61%); WSR test, *N* = 25, *z* = 0.59, *p* = 0.554). An additional ANOVA on the transformed error rates yielded results consistent with the WSR tests, showing a significant interaction effect, but no main effects of either factor.

#### 3.2.2. Response times

In situations where Bayesian updating is aligned with reinforcement (the Right Urn situations), as expected, responses were slower in case of conflict between Bayesian updating and inertia (median 878 ms, mean 1025 ms, *SD* = 503 ms) than in case of alignment (median 666 ms, mean 905 ms, *SD* = 698 ms). The WSR test was significant (*N* = 44, *z* = 3.76, *p* < 0.001). This result holds for both forced draws (conflict: median 943 ms, mean 1136 ms, *SD* = 530 ms; alignment: median 744 ms, mean 961 ms, *SD* = 702 ms; WSR test, *N* = 43, *z* = 2.98, *p* = 0.003) and free draws (conflict: median 796 ms, mean 1031 ms, *SD* = 927 ms; alignment: median 623 ms, mean 896 ms, *SD* = 1141 ms; WSR test, *N* = 43, *z* = 3.48, *p* < 0.001). An additional ANOVA on log-transformed data yielded results consistent with the WSR tests, showing a significant main effect of conflict with vs. alignment with inertia and a significant main effect of forced vs. free draw, but no significant effect of interaction.

In situations where Bayesian updating conflicts with reinforcement (the Left Urn situations), there is no significant difference between the response times in case of conflict between Bayesian updating and inertia (median 1579 ms, mean 1919 ms, *SD* = 1237 ms) and the response times in case of alignment (median 1595 ms, mean 2123 ms, *SD* = 1449 ms; WSR test, *N* = 44, *z* = −0.70, *p* = 0.484). The same result holds when the test is made conditional on free draws (conflict: median 926 ms, mean 1543 ms, *SD* = 1295 ms; alignment: median 1019 ms, mean 1376 ms, *SD* = 770 ms; WSR test, *N* = 25, *z* = 0.58, *p* = 0.563). In forced draws, the results showed that the response times in case of conflict with inertia (median 1671 ms, mean 1971 ms, *SD* = 1438 ms) were faster than in case of alignment with inertia (median 2182 ms, mean 2427 ms, *SD* = 1347 ms; WSR test, *N* = 25, *z* = −2.19, *p* = 0.028). An additional ANOVA on the transformed error rates yielded results consistent with the WSR tests, showing a significant main effect of forced vs. free draw, but no main effect of conflict vs. alignment with inertia and no interaction.

### 3.3. Discussion

In decision situations without additional conflict due to reinforcement, which are comparable to Study 1, the results show that more errors and slower responses are made in case of conflict between Bayesian updating and decision inertia in both forced and free draws, confirming that decision inertia exists for both required and autonomous choices. This replicates the results of Study 1. When decisions are made in the presence of a conflict between Bayesian updating and reinforcement, our results suggest that decision inertia is only present for the case of voluntary (autonomous) previous choices, and even in that case evidence on response times is inconclusive. Our interpretation is that decision inertia is a subtle process, which might be partially washed out when reinforcement conflicts with Bayesian updating.

## 4. Decision inertia and preference for consistency

We now investigate the proposed relationship between inertia and preference for consistency (PFC). At the same time, based on the insights from Studies 1 and 2, we examine more closely whether the effect of decision inertia varies according to the type of decisions (forced vs. free). We measured PFC through the corresponding scale after the decision-making part of the experiment was completed. The average PFC score in our data was 4.28 (*SD* = 1.86). Internal consistency as measured by Cronbach's alpha was 0.83. To uncover the associations between decision inertia and PFC, and between decision inertia and decision autonomy, we ran random-effects probit regressions on second-draw errors for the data from both studies (see Table 2). These regressions allow us to control for a variety of other variables like round number, counterbalancing, and conflict with reinforcement (in Study 2). The results show that in both studies, the interaction effect of conflict with inertia and the PFC score is significantly positive, indicating that in case of conflict with inertia, a higher PFC score is associated with an increased probability of errors, which is consistent with our assumption. In addition, in both studies, the interaction effect of conflict with inertia and forced draws is significantly negative, that is, the effect of decision inertia is stronger in free draws than in forced draws.

**Table 2:** **Random-effects probit regressions on second-draw errors (1 = error) in Studies 1 and 2**.

**Variable**	**Study 1**				**Study 2**			
	**(1)**		**(2)**		**(1)**		**(2)**	
	**β(*SE*)**	***p***	**β(*SE*)**	***p***	**β(*SE*)**	***p***	**β(*SE*)**	***p***
ConflictR(1 = Yes)					2.33(0.09)^***^	< 0.001	2.43(0.10)^***^	< 0.001
ConflictI(1 = Yes)	0.19(0.17)	0.27	0.34(0.18)^*^	0.07	−0.34(0.22)	0.11	0.24(0.24)	0.32
PFC	−0.01(0.07)	0.92	−0.01(0.07)	0.92	0.11(0.06)^*^	0.08	0.11(0.07)	0.10
TrialNr	−0.31(0.12)^***^	0.007	−0.31(0.12)^***^	0.007	−0.30(0.13)^**^	0.02	−0.25(0.13)^*^	0.06
Cb	−0.38(0.24)	0.12	−0.37(0.24)	0.13	0.16(0.20)	0.43	0.16(0.21)	0.44
ConflictI × PFC	0.11(0.04)^***^	0.005	0.11(0.04)^***^	0.005	0.13(0.04)^***^	0.004	0.14(0.04)^***^	0.002
Forced(1 = Yes)			0.20(0.11)^*^	0.06			0.54(0.12)^***^	< 0.001
ConflictI × Forced			−0.29(0.14)^**^	0.035			−1.03(0.16)^***^	< 0.001
Constant	−1.23(0.31)^***^	< 0.001	−1.34(0.31)^***^	< 0.001	−2.84(0.34)^***^	< 0.001	−3.27(0.37)^***^	< 0.001
Wald chi2	97.54^***^	< 0.001	100.56^***^	< 0.001	617.22^***^	< 0.001	606.42^***^	< 0.001
Log likelihood	−954.66		−952.37		−780.66		−759.57	
No. of Obs.	2700		2700		2640		2640	

*ConflictR is a dummy referring to conflict between Bayesian updating and reinforcement. ConflictI is a dummy referring to conflict between Bayesian updating and inertia. Cb is the counterbalance dummy*.

* *p < 0.10

** p < 0.05

*** *p < 0.001*.

## 5. General discussion

This study shows that decision inertia plays a role in human decision making under risk and investigates the underlying processes. We find a significant tendency to repeat previous choices in decision making with monetary feedback. Specifically, we found evidence for the existence of decision inertia in Study 1 and in decision situations without conflict with reinforcement in Study 2. In contrast, in the Left-Urn situations in Study 2, where reinforcement conflicts with Bayesian updating, we only found an effect of decision inertia after autonomous choices. We conclude that decision inertia seems to be subtle and easily overshadowed by stronger processes as e.g., reinforcement learning.

We hypothesized that decision inertia would be positively associated with PFC. The regression analysis confirms this hypothesis, indicating that the tendency to repeat past choices is a relevant part of the need to be consistent. This finding agrees with those of Pitz (1969), who showed that the inertia effect in opinion revision results from a psychological commitment to one's initial judgments. It is not consistent with the results of Zhang et al. (2014, Study 2b), who found no relation between repetition of earlier decisions and PFC scores. However, Zhang et al. (2014) targeted unethical decisions and hence their setting is hard to compare to ours. The moral framing of the decisions in that work might have interacted with the hypothesized need for consistency. Our results for free vs. forced draws provide further evidence that decision inertia might (at least partly) be based on a mechanism of consistency-seeking. Both of our studies suggest that the effect of decision inertia might vary according to the type of first-draw decisions. The results of the regression analyses confirm this idea, indicating that decision inertia is significantly stronger in autonomous choices than in required ones. Since one would assume that a psychological desire to be consistent with one's own decisions is stronger for self-selected compared to assigned decisions, this result further supports an interpretation of decision inertia as a facet of consistency-seeking.

Our results are also in agreement with the reinforcement learning literature (e.g., Schönberg et al., 2007; Gershman et al., 2009; Wimmer et al., 2012) which has pointed out the importance of perseveration as an additional factor. A direct comparison is of course difficult, because in our paradigm success probabilities are explicitly given (and priors are reset after every round), while in the quoted works they are discovered through experience. However, the basic messages are similar. As in those previous reports, we find that the mere repetition of previous choices plays a role even when behavior is mostly determined by the interaction of reinforcement and normative goals. In that sense, we confirm (in a different setting) that incorporating perseveration into models of reinforcement learning can improve our understanding of how errors occur.

In conclusion, we find clear evidence for the existence of decision inertia in incentivized decision making. Our study sheds light on the process underlying decision inertia, by showing that this behavioral tendency is positively associated with an individual's preference for consistency, and that the effect of decision inertia is stronger in voluntary choices than in required choices.

## Author contributions

All authors contributed equally to this work. The listing of authors is alphabetical.

### Conflict of interest statement

The authors declare that the research was conducted in the absence of any commercial or financial relationships that could be construed as a potential conflict of interest.
